# Supplementary material for: Light-Regulated Transcription of a Mitochondrial-Targeted K+ Channel
Source: Cells. 2020 Nov 19;9(11):2507. doi: 10.3390/cells9112507 (PMC7699372; doi:10.3390/cells9112507)
Supplement: Supplementary file 1 [file cells-09-02507-s001.pdf]

Supplementary Figures

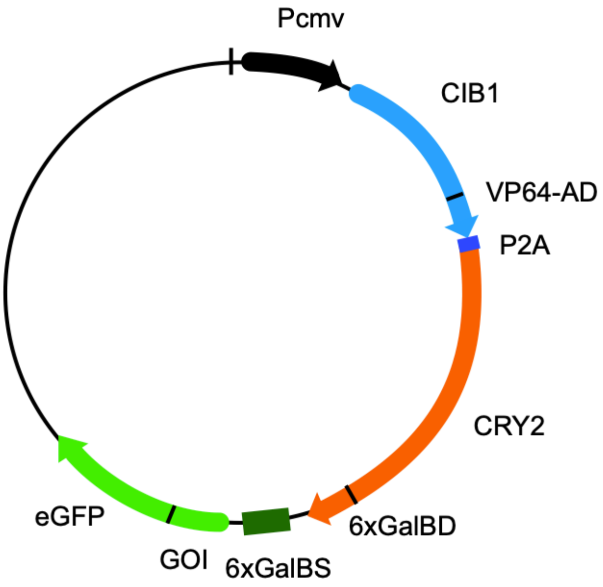

Figure S1. Organization of unified vector. The vector includes a CMV promotor (Pcmv), a fusion of CIB1 with the VP64 activation domain and CRY2 separated by a self-cleaving P2A sequence. The pGL2-Gal4-GOI which codes for the GOI which is fused to a 6xGalBS and has an c-terminal eGFP-tag.

A

```
Kmpv12T  ------------------------------------------MRNLIIISTLFGIIY----S    17
Kesv     MSRRLFATCGIAIALRGLVVSGGVKEIVSFRPLIDTSLVGGILSNLIL-LVVFAELYWQLDQ    61
                                                   : ***:  .:*. :*    .

Kmpv12T  SLEPGHFQFKSVLDPFYFSFTTMSSVGYGDITPKTNLAKVLVMCQQSLLFNELMQ-VAKMIKY   78
Kesv     GDDHTHFGFSSAIDAYYFSAVTSSSVGYGDLLPKTPKAKLLTIAHILAMFFVMLPVVAKALEK   124
         . :  ** *.*.:* :*** .* *******: ***  **:*.:.:   :*  ::  *** ::
```

B

Kesv

ATGAGCAGACGGCTGTTCGCCACCTGTGGAATCGCCATTGCCCTGCGGGGCCTGGTGGTGTCTGGCGGCGTGAAA
GAAATCGTGTCCTTCCGGCCCCTGATCGACACCAGCCTCGTGGGAGGCATCCTGAGCAACCTGATCCTGCTGGTG
GTGTTCGCCGAGCTGTACTGGCAGCTGGACCAGGGCGACGACCACACCCACTTCGGCTTCAGCAGCGCCATCGAC
GCCTACTACTTCAGCGCCGTGACCAGCAGCAGCGTGGGCTACGGCGACCTGCTGCCCAAGACCCCCAAGGCCAAG
CTGCTGACAATCGCCCACATCCTGGCCATGTTCTTCGTGATGCTGCCCGTGGTGGCCAAGGCCCTGGAAAAGG

Kmpv$_{12T}$

ATGCGGAACCTGATCATCATCAGCACCCTGTTCGGCATCATCTACAGCAGCCTGGAACCTGGCCACTTCCAGTTC
AAGAGCGTGCTGGACCCCTTCTACTTCAGCTTCACCACCATGAGCAGCGTCGGCTACGGCGACATCACCCCTAAG
ACCAATCTGGCCAAGGTGCTGGTCATGTGCCAGCAGAGCCTGCTGTTCAACGAGCTGATGCAGGTCGCCAAGATG
ATCAAGTAC

Figure S2: Sequences of viral K^+ channels. Two viral encoded proteins with hallmarks of K^+ channels. **(A)** Alignment of amino acid sequences of Kesv and Kmpv$_{12T}$. The typical sequence of the selectivity filter is highlighted in red. **(B)** Sequences of codon optimized genes of both proteins.

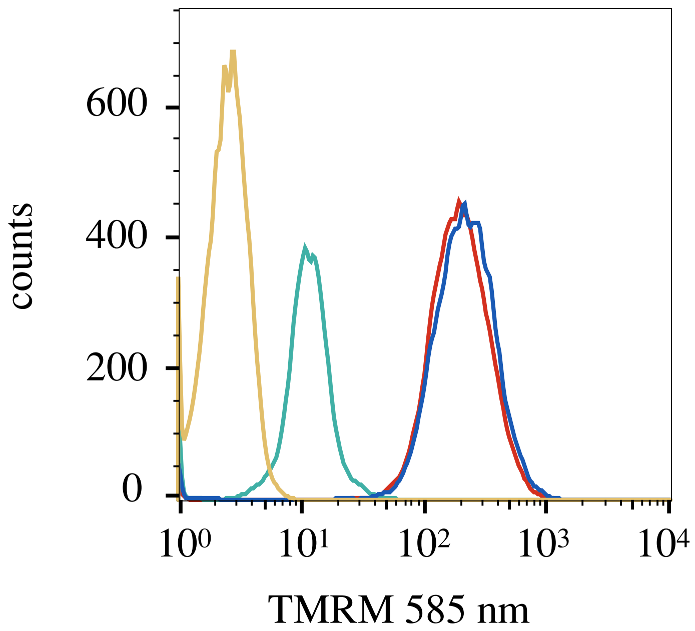

Fig. S3: Kesv does not depolarize mitochondrial membrane potential.
Spectra of unstained HEK293 cells (yellow) and TMRM stained un-transfected control cells (blue) and cells expressing Kesv without (red) or with 100 µM CCCP (green).

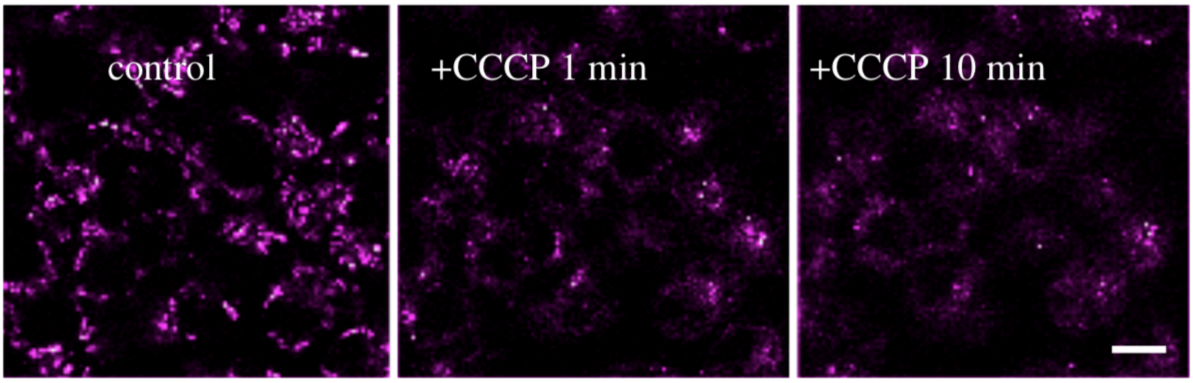

Figure S4: Uncoupler CCCP causes a decrease in MitoTracker CMXRos fluorescence. Hek293 cells were stained with voltage sensitive mitotracker dye CMXRos, which resulted in bright fluorescence of mitochondria (left panel). Addition of 100 µM CCCP to buffer resulted in strong decrease in CMXRos fluorescence 1 min and 10 min after addition of uncoupler. The effect of the uncoupler can be quantified from the ratio of red fluorescence from cells in CCCP (RFI_{+CCCP}) divided by fluorescence from the same cells prior to CCCP treatment (RFI_{-CCCP}).

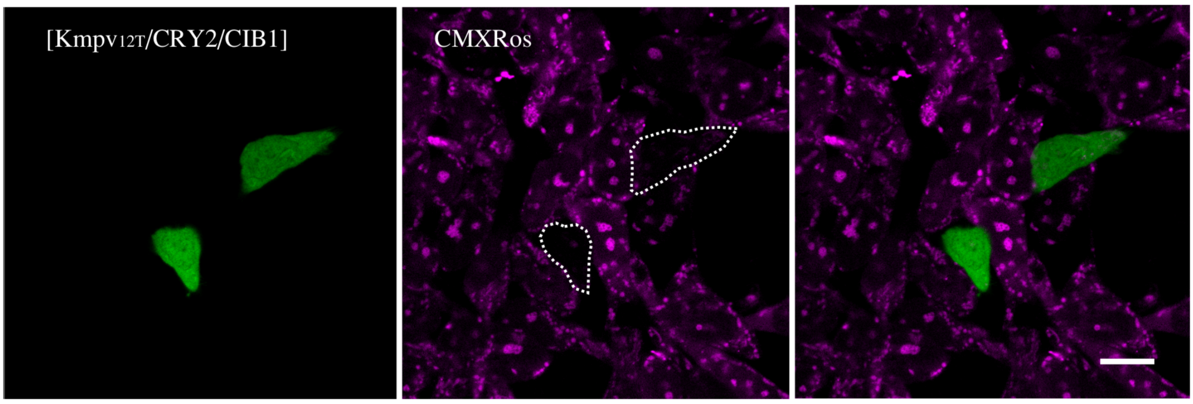

Figure S5: Also Kmpv$_{12T}$ with no distinct mitochondrial sorting causes depolarization of mitochondria. Fluorescent images of HEK293 cells expressing **[**Kmpv$_{12T}$/CRY2/CIB1**]** with GFP throughout entire cell (left panel) and MitoTracker CMXRos (2nd column A). Overlays of green and CMXRos fluorescence are reported in third column. The contours of GFP positive cells in red channels are indicated by dashed lines. The red fluorescence in the GFP positive cells is lower than in the adjacent non transfected cells. Cells were incubated for 16 hours and illuminated with a single 1-minute light pulse of 6 μmol 4 hours prior to imaging.

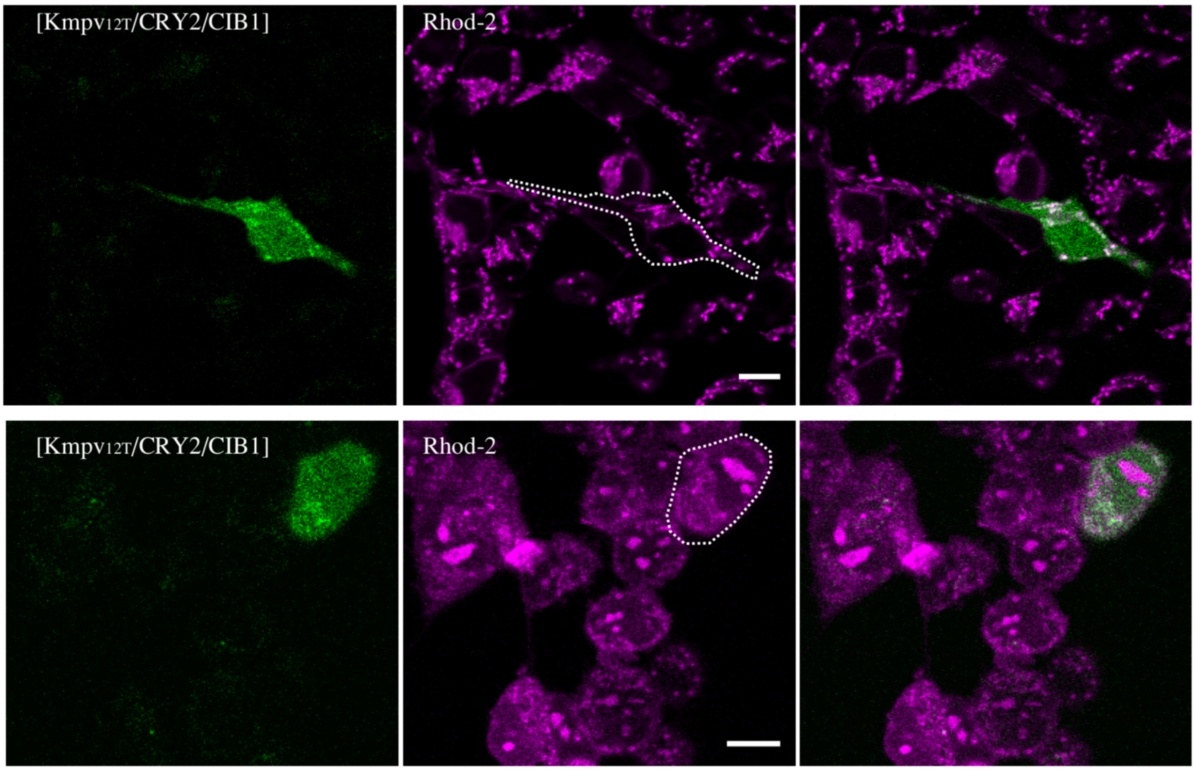

Figure S6: Expression of [Kmpv$_{12T}$/CRY2/CIB1] in the dark does not affect fluorescence of voltage sensor CMXRos or Ca^{2+} sensor Rhod-2. Fluorescent images of HEK293 cells expressing GFP tagged CRY-Kmpv$_{12T}$ (1st column) and stained (2nd column) with MitoTracker CMXRos (top row) or Rhod-2 (bottom row) and overlay of GFP with CMXRos or Rhod-2 fluorescence (3rd column). The contours of GFP positive cells in red channels are indicated by dashed lines. The GFP signal is very low because Kmpv$_{12T}$ expression was under control of CRY2/CIB1 system and cells were kept in the dark. In the latter case cells were incubated for 20 hours in dark prior to imaging. Calibration bars= 10 μm.
